# Supplementary material for: Loss of mitochondrial enzyme GPT2 leads to reprogramming of synaptic glutamate metabolism
Source: Mol Brain. 2024 Nov 27;17:87. doi: 10.1186/s13041-024-01154-x (PMC11600823; doi:10.1186/s13041-024-01154-x)
Supplement: Supplementary file 1 — Supplementary Material 1. [file 13041_2024_1154_MOESM1_ESM.pdf]

Supporting Information

Loss of mitochondrial enzyme GPT2 leads to reprogramming of synaptic glutamate metabolism

Running Title: Loss of GPT2 reprograms synaptic glutamate metabolism

Ozan Baytas, Shawn M. Davidson, Julie A. Kauer and Eric M. Morrow

Supporting Information for this article includes 6 supporting figures:

- **Figure S1.** VGLUT1 and VGAT protein levels are increased in *Gpt2*-null synaptosomes.
- **Figure S2.** Aspartate aminotransferase and glutaminase protein levels and enzyme activities in *Gpt2*-null synaptosomes.
- **Figure S3.** Asymmetric spine and mitochondria counts are unchanged in electron micrographs of CA1 stratum radiatum of *Gpt2*-null hippocampus.
- **Figure S4.** Alanine and alpha-ketoglutarate readily enter synaptosomes.
- **Figure S5.** Glutamine entry and nitrogen labeling of glutamine and glutamate in *Gpt2*-null synaptosomes.
- **Figure S6.** Confirmation of glutamate dehydrogenase enzyme activity in synaptosomes.

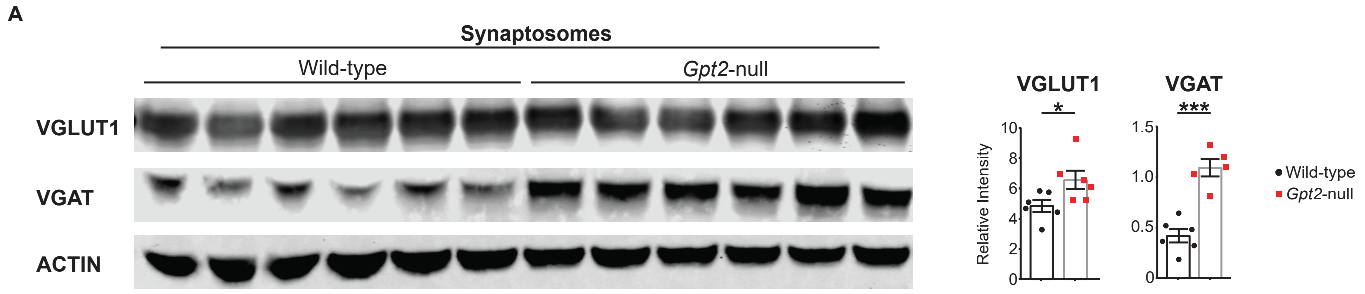

Figure S1. VGLUT1 and VGAT protein levels are increased in Gpt2-null synaptosomes.

A. Western blotting of wild-type and Gpt2-null synaptosome protein lysates collected at P18. Each dot represents a different protein lysate sample from wild-type (black) or Gpt2-null (red) mice. Each protein band is normalized to its corresponding intensity of the actin band. Wild-type vs. Gpt2-null: *P (VGLUT1, vesicular glutamate transporter 1) = 0.038; ***P (VGAT, vesicular GABA transporter) = 0.0001.

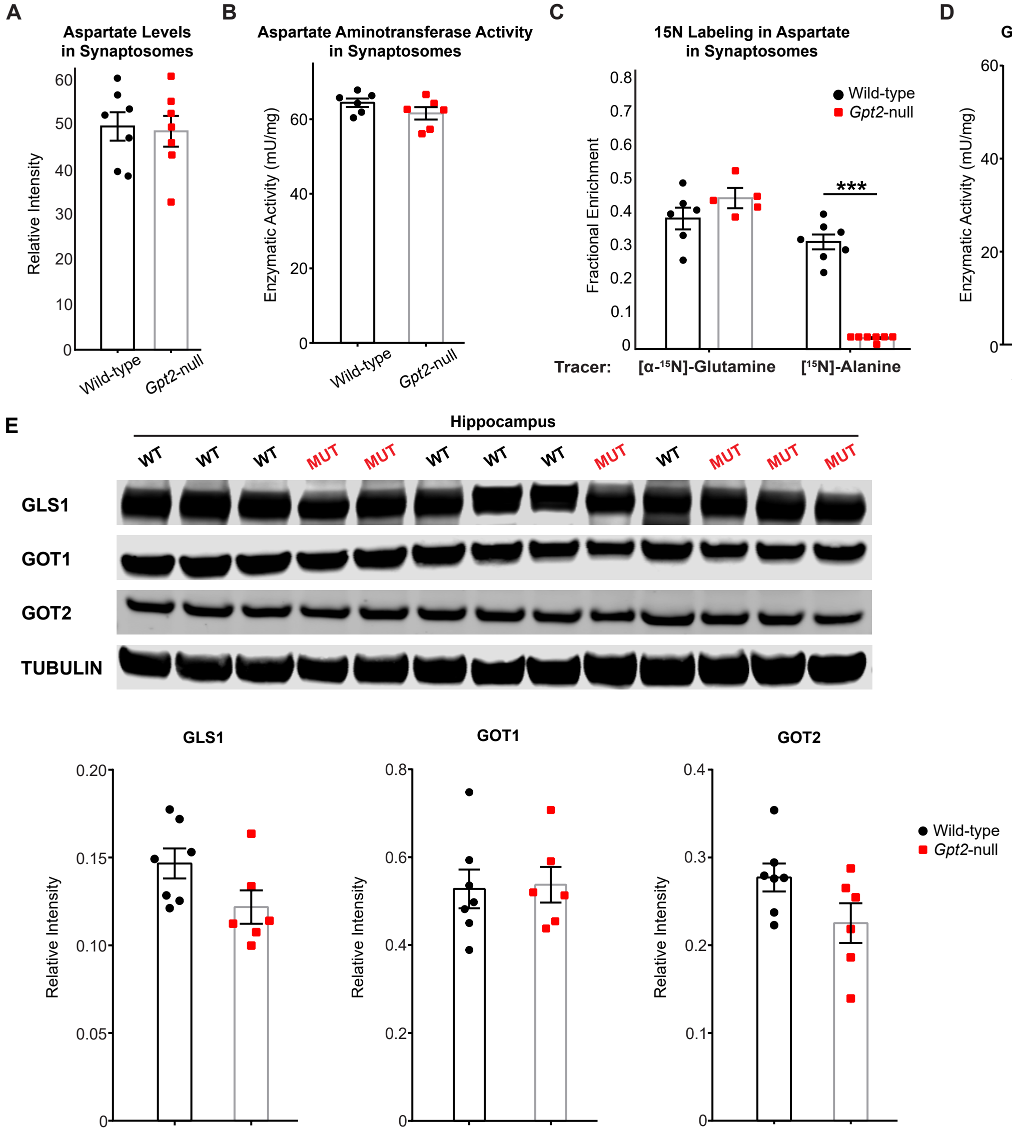

Figure S2. Aspartate aminotransferase and glutaminase enzyme activities in *Gpt2*-null synaptosomes.
A. Aspartate levels in *Gpt2*-null synaptosomes. Each dot represents a different synaptosome sample obtained from wild-type (black) or *Gpt2*-null (red) mice at P18.
B. Aspartate aminotransferase enzyme activity in wild-type and *Gpt2*-null synaptosomes. Each dot represents a different synaptosome sample obtained from wild-type (black) or *Gpt2*-null (red) mice at P18.
C. 15N (heavy nitrogen) labeling of aspartate in *Gpt2*-null synaptosomes using [α-15N]-glutamine or [α-15N]-alanine as tracers. Each dot represents a different synaptosome sample obtained from wild-type (black) or *Gpt2*-null (red) mice at P18. ****P* <0.0001.
D. Glutaminase enzyme activity in wild-type and *Gpt2*-null synaptosomes. Each dot represents a different synaptosome sample obtained from wild-type (black) or *Gpt2*-null (red) mice at P18.
E. Western blotting for aspartate aminotransferase 1 (GOT1), aspartate aminotransferase 2 (GOT2) and glutaminase (GLS1) in wild-type and *Gpt2*-null hippocampus protein lysates. Each dot represents a different synaptosome sample obtained from wild-type (black) or *Gpt2*-null (red) mice at P18. Each protein band is normalized to its corresponding intensity of the tubulin band.

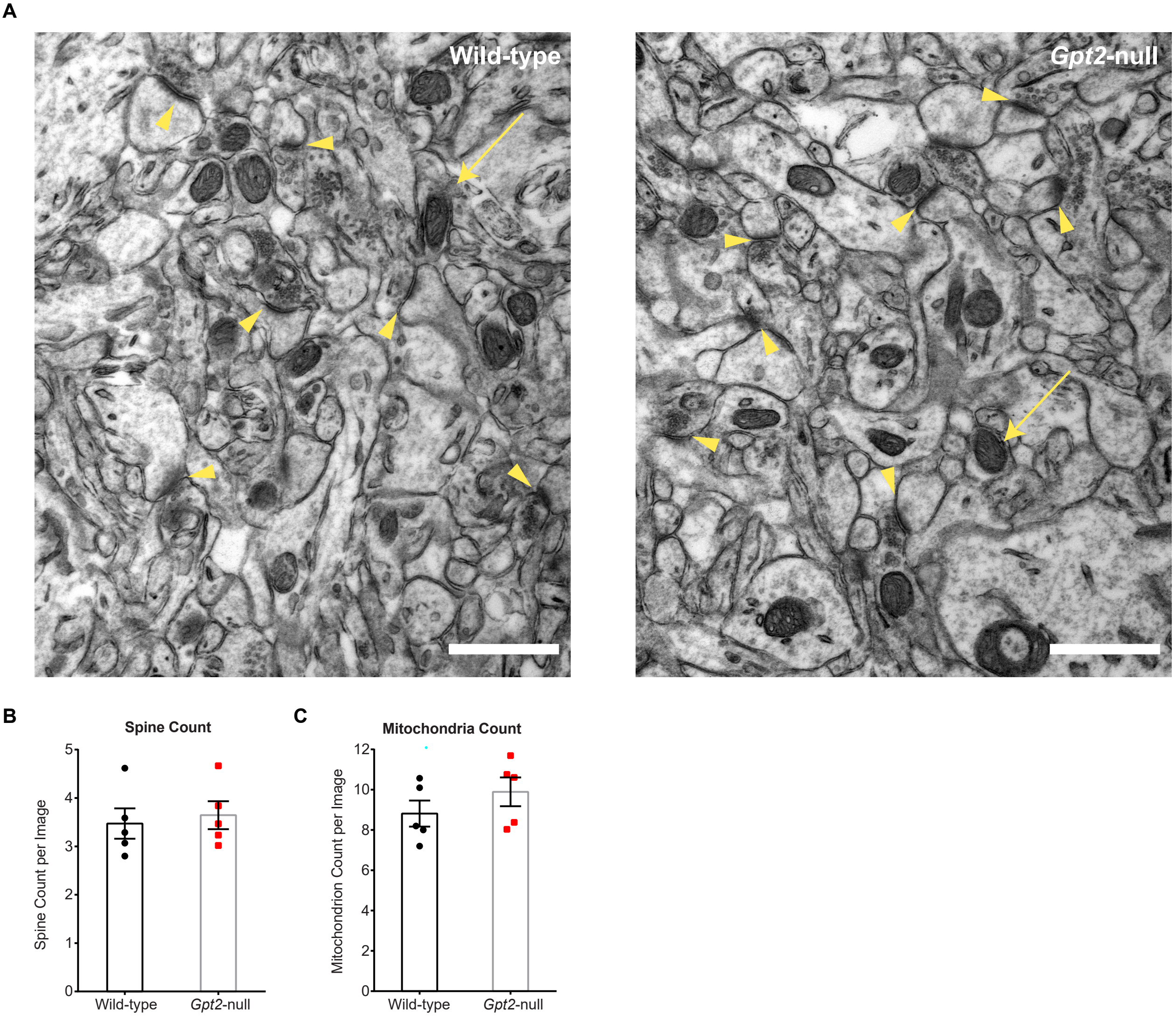

Figure S3. Asymmetric spine and mitochondria counts are unchanged in electron micrographs of CA1 stratum radiatum of *Gpt2*-null hippocampus.

A. Representative electron micrographs of wild-type (left) and *Gpt2*-null (right) CA1 stratum radiatum of the hippocampus at P18. Orange arrowheads point to the asymmetric spines, arrows point to the mitochondria. Scale bar: 1 µm.

B. Quantification of spines per image (21000X magnification). Each dot represents the averaged number of spines obtained from one animal. More than 25 micrographs from each animal were analyzed.

C. Quantification of mitochondria per image (21000X magnification). Each dot represents the averaged number of mitochondria obtained from one animal. More than 25 micrographs from each animal were analyzed.

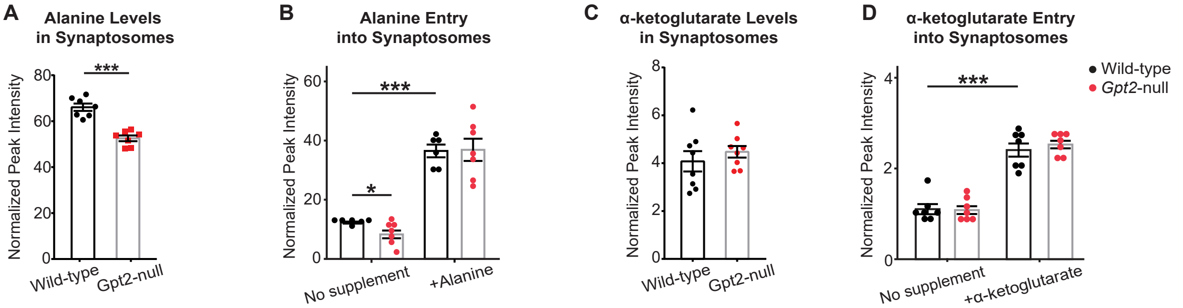

Figure S4. Alanine and alpha-ketoglutarate readily enter synaptosomes.
A. Alanine levels are reduced in *Gpt2*-null synaptosomes. Each dot represents a different synaptosome sample obtained from wild-type (black) or *Gpt2*-null (red) mice at P18. ***P <0.0001.

B. Alanine can readily enter the synaptosomes. Each dot represents a different synaptosome sample obtained from wild-type (black) or *Gpt2*-null (red) mice at P18. No supplement: wild-type vs. *Gpt2*-null: *P = 0.02. Wild-type no supplement vs. wild-type +alanine: ***P <0.0001. +alanine: wild-type vs. *Gpt2*-null: P = 0.94.

C. Alpha-ketoglutarate levels are not changed in *Gpt2*-null synaptosomes. Each dot represents a different synaptosome sample obtained from wild-type (black) or *Gpt2*-null (red) mice at P18. P = 0.43.

D. Alpha-ketoglutarate can readily enter the synaptosomes. Each dot represents a different synaptosome sample obtained from wild-type (black) or *Gpt2*-null (red) mice at P18. Wild-type no supplement vs. wild-type +alpha-ketoglutarate: ***P <0.0001.

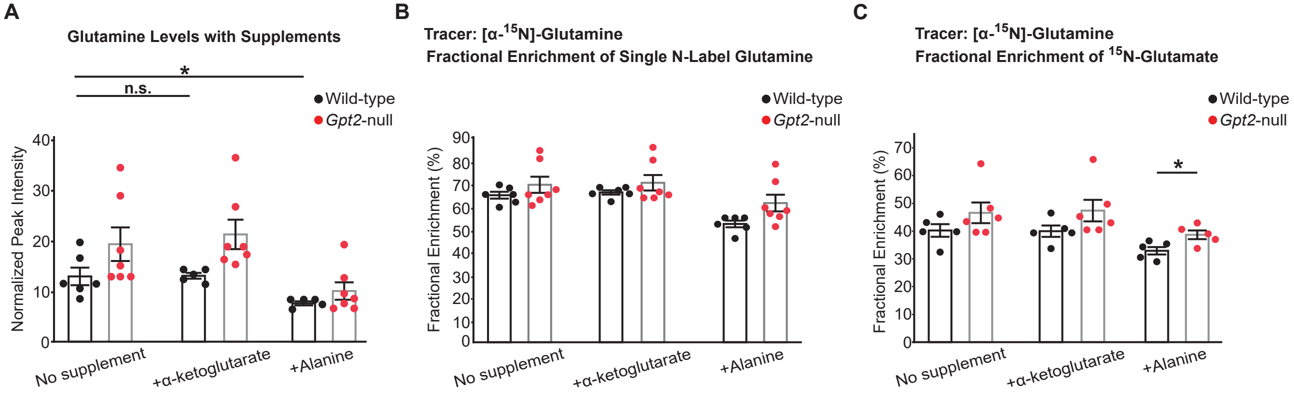

Figure S5. Glutamine entry and nitrogen labeling of glutamine and glutamate in *Gpt2*-null synaptosomes.

A. Entry of glutamine is unaffected by alpha-ketoglutarate. Each dot represents synaptosome samples obtained from different wild-type (black) or *Gpt2*-null (red) mice at P18. Wild-type no supplement vs. +alanine: *$P = 0.02$; Wild-type no supplement vs. +alpha-ketoglutarate: $P = 0.95$.

B. Exogenous amine labeled glutamine labels the majority of glutamine pool in synaptosomes. Each dot represents synaptosome samples obtained from different wild-type (black) or *Gpt2*-null (red) mice at P18. Wild-type vs. *Gpt2*-null no supplement: $P = 0.29$; +alanine: $P = 0.0513$; +alpha-ketoglutarate: $P = 0.29$.

C. Fractional enrichment of labeled glutamate with the amine heavy nitrogen labeled glutamine precursor. Each dot represents synaptosome samples obtained from different wild-type (black) or *Gpt2*-null (red) mice at P18. Wild-type vs. *Gpt2*-null no supplement: $P = 0.2$; +alanine: *$P = 0.023$; +alpha-ketoglutarate: $P = 0.15$.

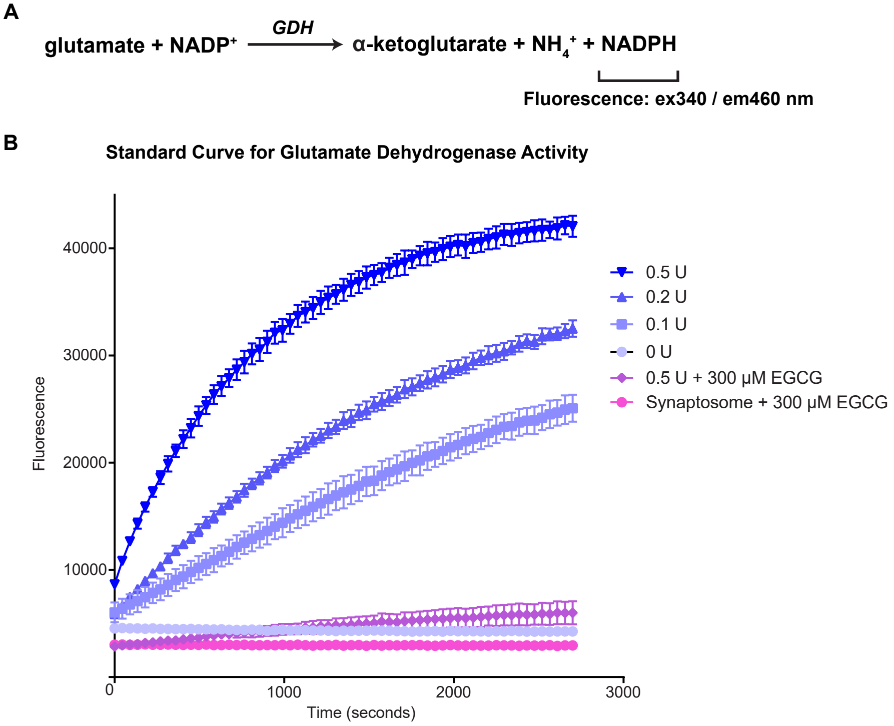

Figure S6. Confirmation of glutamate dehydrogenase enzyme activity in synaptosomes. **A.** Chemical reaction catalyzed by glutamate dehydrogenase (GDH). The enzyme activity assay detects fluorescence with excitation and emission wavelengths of 340 and 460 nm, respectively.

B. Standard curve used to determine GDH enzymatic activity in synaptosomes. 300 μM Epigallocatechin gallate (EGCG) is used as a glutamate dehydrogenase inhibitor to validate the assay [76].
